# Differential impact of transfusion guidelines on blood transfusion practices within a health network

**DOI:** 10.1038/s41598-023-33549-6

**Published:** 2023-04-17

**Authors:** Spyros Balafas, Vanessa Gagliano, Clelia Di Serio, Giuglia Andrea Guidugli, Andrea Saporito, Luca Gabutti, Paolo Ferrari

**Affiliations:** 1 University Centre of Statistics in the Biomedical Sciences CUSSB, UniSR, Milan, Italy; 2 Vita-Salute San Raffaele University, UniSR, Milan, Italy; 3 Department of Internal Medicine, Ente Ospedaliero Cantonale, Bellinzona, Switzerland; 4 Faculty of Biomedicine, Università Della Svizzera Italiana, Lugano, Switzerland; 5 Division of Anesthesiology, Ente Ospedaliero Cantonale, Bellinzona, Switzerland; 6 Clinical School, University of New South Wales, Sydney, Australia; 7 Department of Nephrology, Ente Ospedaliero Cantonale (EOC), 6900 Lugano, Switzerland

**Keywords:** Health policy, Public health, Anaemia

## Abstract

Whether clinical practice guidelines have a significant impact on practice is unclear. The effect of guideline recommendations on clinical practice often a lags behind the date of publication. We evaluated by means of a data-driven approach if and when the guidelines on red blood cell transfusions (RBCTs) issued by Swiss Smarter Medicine in 2016 had an impact on RBCTs practice within a hospital network, where awareness of guidelines was promoted mainly among internal medicine specialties. Data on RBCTs performed in a Swiss hospital network from January 2014 to April 2021 were analyzed by hospital site and specialty to assess whether guidelines led to a decrease in inappropriate RBCTs. RBCTs were defined as “inappropriate” if patients had a hemoglobin level ≥ 70 g/L without or ≥ 80 g/L with significant cardiovascular comorbidities. Changes in the rate of inappropriate transfusions were analyzed with an advanced statistical approach that included generalized additive models. Overall prior to March 2017 there were more inappropriate than appropriate RBCTs, but after October 2017 the opposite could be observed. A change-point in the time trend was estimated from transfusion data to occur in the time interval between March and October 2017. This change was mainly driven by practice changes in the medical wards, while no significant change was observed in the critical care, surgical and oncology wards. Change in practice varied by hospital site. In conclusion, our results show that a significant change in the RBCTs practice at the hospital level occurred approximately 18 months after national guidelines were issued.

## Introduction

Red blood cells transfusions (RBCTs) are routinely used for the management of severe anemia and bleeding. Annually, more than 100 million units of blood are collected worldwide^1^ and RBCTs are one of the five most overused procedures in hospitals^2^. Despite being considered generally safe, the administration of allogenic RBCTs is an independent risk factor for morbidity and mortality^3–7^. Therefore, reducing the unnecessary use of RBCTs is important both for patient outcomes and to optimize use of a limited resource^8^. Safe RBCT practices depend on the match between the right indication, the right amount of RBCTs, and the right timing. Recent evidence has shown that restrictive transfusion thresholds are at least as effective as liberal transfusion thresholds^8^ and that they do not correlate with increased morbidity or mortality in different settings^9^. In addition, it is now generally agreed to administer only one RBCT unit at first, and to consider giving a second unit based on patient symptoms and Hb course^10^.

In order to reduce the rate of inappropriate RBCTs, a number of interventions, such as audit and feedback, clinicians’ education, organizational policy change, and clinical decision support tools, have been endeavored^11^. In 2012, the American Board of Internal Medicine Foundation instituted the Choosing Wisely campaign, aiming at sensitizing health care professionals towards a better use of medical resources at the national level. The same year, the American Association of Blood Banks (AABB) guidelines^12^ first suggested maintaining a restrictive transfusion strategy with a hemoglobin (Hb) threshold of 70 to 80 g/L for stable hospitalized patients, and ≤ 80 g/L for symptomatic patients with pre-existing cardiovascular disease^13^. The same year, the Australian Patient Blood Management (PBM) guidelines^14^ advised to initiate transfusion at Hb < 70 g/L even in patients with heart failure and at < 80 g/L in those with an acute coronary syndrome, while in 2013 the American Society of Hematology (ASH) released a recommendation similar to that of AABB^15^. Later, in 2015, the National Institute for Health and Care Excellence (NICE) guideline^10^ reinforced the concept of keeping the Hb transfusion threshold at 80 g/L for patients with ongoing acute coronary syndrome, and at 70 g/L for all the other cases, in the absence of a major hemorrhage, while in 2016 the Swiss Society of General Internal Medicine (SSGIM) released a recommendation^11^, similar to that of ASH. Thereafter, under the umbrella of the SSGIM a number of awareness-raising events were organized throughout the country for the purpose of bridging the gap between theory and clinical practice, while providing useful reference points for both the patients and their treating physicians and caregivers.

Prescription habits following publication of guidelines are mainly influenced if supported by multimodal interventions including educational material and awareness campaign and it often takes years for them to shift practice. With awareness campaigns we observed some temporary improvements in mitigating overtreatment^16,17^, however, these measures appear insufficient in creating a lasting change^18,19^. Following the release of the SSGIM recommendations on RBCTs in May 2016, awareness on the new guidelines was promoted within our hospital network mainly within the department of internal medicine, but not other specialties.

Thus, the aim of the present study was to understand the actual time lag between the introduction of new guidelines and their actual adoption in everyday clinical practice, in order to better design and organize the implementation of future healthcare policies. We therefore implemented a data-driven approach that allows to identify in which time-window a statistical significant “change-point” occurs, thus allowing to evaluate the real effect of publications of guidelines and in-house awareness campaign in relation to specialty and hospital site.

### Study design and methods

We conducted a retrospective analysis of the number and appropriateness of RBCTs administered to all patients who accessed the four specialties of internal medicine (MED), surgery (SURG), oncology (ONC) and intensive care (ICU) at the public hospital network of Ente Ospedaliero Cantonale (EOC) in Southern Switzerland between January 2014 and April 2021. The network involved 4 acute care hospitals. All methods were performed in accordance with the relevant guidelines and regulations. Deidentified data was extracted from the EOC’s Patient Blood Management (PBM) Data System, which was created in April 2021 to support clinicians in the implementation of transfusion guidelines. The EOC PBM Data System collects epidemiological, clinical, and economic patient-level data from the electronic medical, laboratory and administrative records, and hospital coding and billing systems. Data are updated daily and are visualized and reported via linked dashboards. The dashboards allow users to select, filter, and extract data, providing real-time trends of key indicators, such as total transfusion rate, transfusion index, rate of inappropriate transfusions, and length of hospital stay, unexpected readmissions and total costs per case. The dashboard organizes data in ranges, graphs as well as tables, which visualize trends, and has filters to sort data by hospital and/or department and/or type of procedure.

Transfusions were defined as inappropriate if RBCTs were administered either (a) to a patient without significant cardiovascular comorbidity (CVC) and Hb ≥ 70 g/L, or (b) to a patient with CVC and Hb ≥ 80 g/L at the time of transfusion. Data was analyzed by hospital site (Hospital 1–4) and by medical specialties. The supplier of transfusions to participating hospitals was the regional blood bank, there was never a shortage of a timely supply that could have introduced a bias in transfusion strategies throughout the study period.

### Statistical analysis

Statistical modelling of the daily counts for appropriate and inappropriate RBCTs requires a flexible framework that can handle (i) temporal autocorrelation, (ii) time trends with unknown functional forms, and (iii) overdispersion. To tackle these issues simultaneously, we consider a negative binomial Generalized Additive Model (nbGAM)^20^, which is nonparametric regression tool for count data that extends the negative binomial Generalized Linear Model (nbGLM)^21^ by modelling the outcome as a sum of arbitrary functions of the covariates. Each observation in the data was modelled as a function of autoregressive effects, and a time-varying intercept for each level of the interaction between the type of transfusion, hospital, and hospital department. In order to determine the appropriate distribution for the data at hand, we fitted models under a regular Poisson, negative binomial, and zero-inflated Poisson distributions respectively. These models were then used to perform simulation-based tests for overdispersion and zero inflation. From the test results, the nbGAM was the only model among the three that showed no significant overdispersion (dispersion = 0.98547, p-value = 0.272) and no significant zero-inflation (ratioObsSim = 1.0002, p-value = 0.904). Based on these results, the negative binomial distribution was selected as the most appropriate to model the counts of RBCTs. The count time series of RBCTs for each day from 01-01-2014 until 23-04-2021 were modelled using a nbGAM, and they were assumed to be a function of their history from the past week, an intercept for each level of the interaction between type, hospital, and medical specialty, and a smooth effect of time for each level of the interaction between type, hospital, and medical specialty. The analysis in this study was performed in R statistical software using libraries therein. GAMs were fitted by the package **mgcv**^22,23^, and simulation-based tests for overdispersion and zero inflation were conducted using the **DHARMa**^24^ package. For visualization, the R packages **mgcViz**^25^ and **itsadug**^26^ were employed. For more information on the statistical model used for the analysis see Appendix 1.

### Statement of ethics

The study was approved by the local ethics committee (Comitato Etico Cantonale BASEC-2021-00287) and written informed consent was waived as all data was fully anonymized.

## Results

During the observation period a total of 14,715 episodes of RBCTs were observed; 85.8% of patients received 1 unit, 12.7% received 2 units and 1.5% received 3 or more units of RBCTs. In total 18,728 units of red blood cells were given (Table 1). ANOVA analysis of data in Table 1 showed no difference between hospitals and medical specialties, therefore suggesting that the information provided in this study is fully related to the time trends. Hospital 4 had the largest number of RBCTs usage, primarily driven by the demand in the oncology ward. Hospital 1, which is a major surgical and critical care center had the highest use of RBCTs in those two specialties, with 47% of all RBCTs administered in SURG wards and 41% of all RBCTs administered in ICU wards (Table 1). In order to calibrate the data analyzed to hospital activity, we also referred to RBCTs per hospital bed. The analysis on the marginalized data in Table 1 made no difference when related to hospital beds, except for the surgical ward at hospital 1, which is the major trauma center in our hospital network.

**Table 1:** Counts and relative frequencies of red blood cells transfusions across hospitals (columns) and medical specialties (rows) during the observation period.

Beds	Hospital 1 185	Hospital 2 87	Hospital 3 119	Hospital 4 174	Total
Surgery	3149 (48.0%)	893 (40.5%)	810 (25.8%)	1304 (19.1%)	6156 (32.9%)
ICU	1908 (29.1%)	499 (22.6%)	948 (30.2%)	1390 (20.4%)	4745 (25.3%)
Oncology	–	–	–	2849 (41.8%)	2849 (15.2%)
Medicine	1509 (23.0%)	814 (36.9%)	1382 (44.0%)	1273 (18.7%)	4978 (26.6%)
	6566 (35.0%)	2206 (11.8%)	3140 (16.8%)	6816 (36.4%)	18,728 (100.0%)

**Figure 1:**
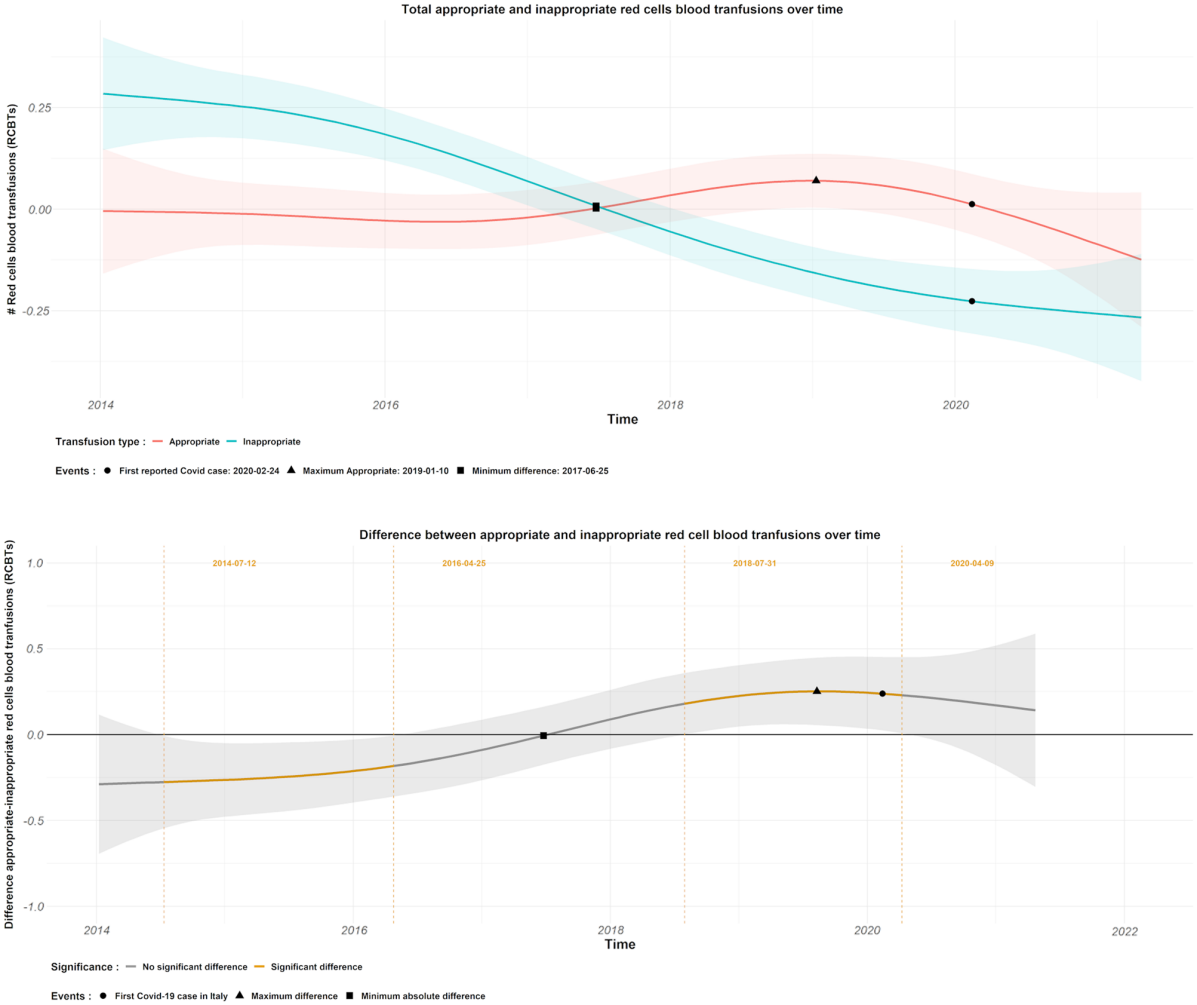
Top panel: Average (across hospitals and medical wards) smooth time trends for appropriate (red) and inappropriate (blue) RBCTs with their corresponding 95% CI. The time of zero difference between the two smooth functions is denoted by a square, the time of maximum appropriate RBCTs with a triangle, and the time of the first COVID-19 reported case in the region by a circle. Bottom panel: Estimated difference (appropriate–inappropriate) with 95% CI between the average smooth time trends of appropriate and inappropriate RBCTs. Time periods of significant difference (zero not included in the 95% CI) between the two smooth components are highlighted in yellow. The timepoint of the maximum difference between the two smooth components is visualized by a triangle.

**Figure 2:**
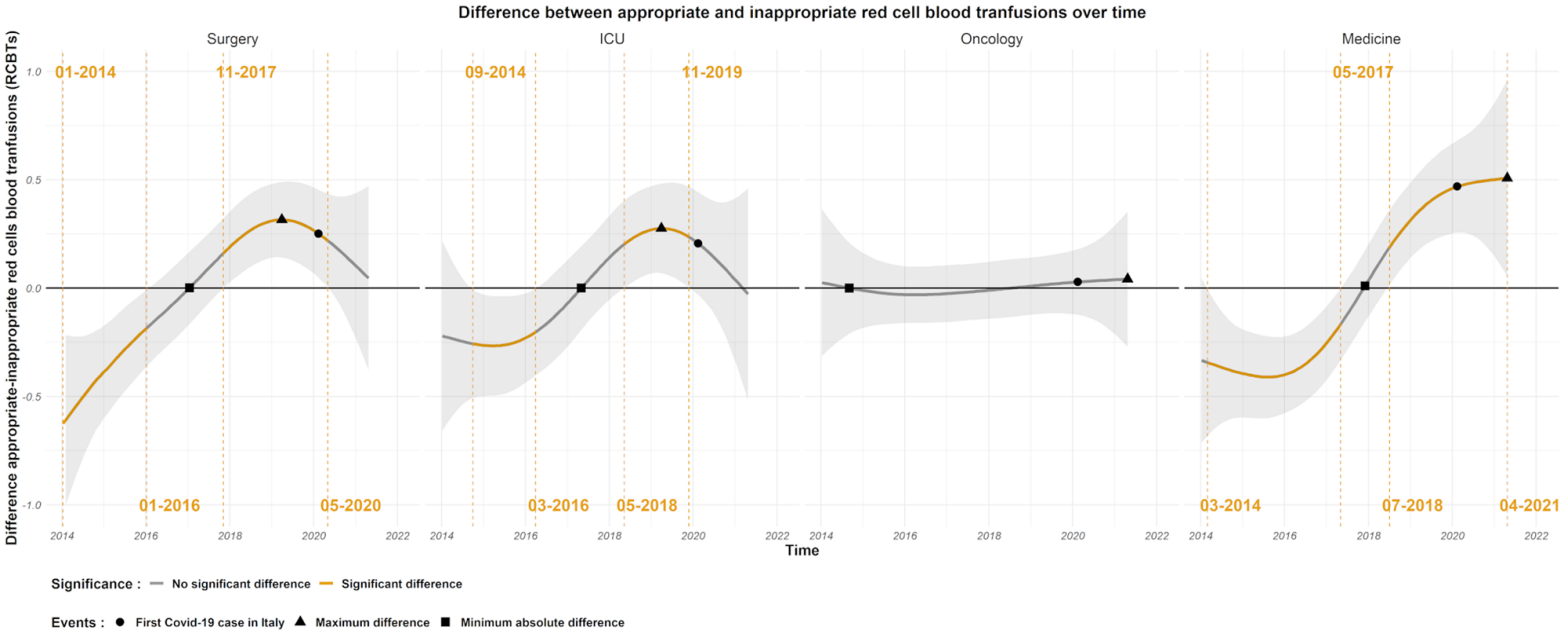
Average (across hospitals) difference in the smooth time trend between appropriate and inappropriate RBCTs in the surgical (SURG), intensive care unit (ICU), oncology (ONC) and medical (MED) wards. Time periods of significant difference (zero not included in the 95% CI) between the two smooth components are highlighted in yellow. The timepoint of the maximum difference between the two smooth components is visualized by a triangle, the time of zero difference by a square, and the first reported COVID-19 case in the region is represented by a circle.

**Figure 3:**
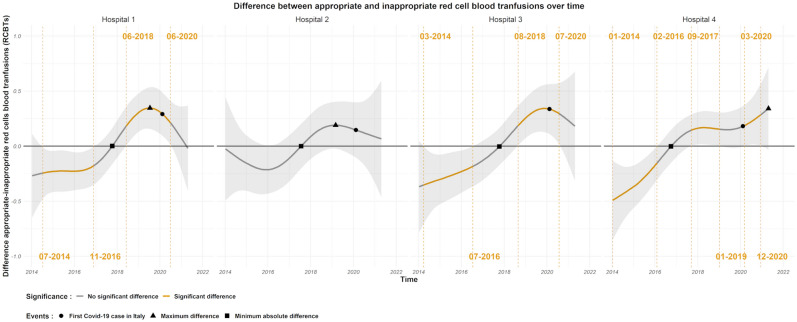
Average (across wards) difference in the smooth time trend between appropriate and inappropriate RBCTs at hospital sites 1 to 4. Time periods of significant difference (zero not included in the 95% CI) between the two smooth components are highlighted in yellow. The timepoint of the maximum difference between the two smooth components is visualized by a triangle, the time of zero difference by a square, and the first reported COVID-19 case in the region is represented by a circle.

Figure 1, top panel shows the average (across hospitals and specialties) smooth time trend for appropriate and inappropriate RBCTs over the observation period and the difference between appropriate and inappropriate RBCTs is shown in Fig. 1, bottom panel. Overall, this difference is significantly different than zero in two different periods. In the first period from July 2014 until April 2016 the inappropriate RBCTs is larger than the appropriate, while between August 2018 and April 2020 the reverse can be observed. A change-point in the time trend is seen between April 2016 and August 2018 (the difference between appropriate and inappropriate RBCTs becomes zero by the end of June 2017). With change-point we refer to a sign change in the difference between appropriate and inappropriate RBCTs. Figure 2 shows the difference over time between appropriate and inappropriate RBCTs in the SURG, ICU, ONC and MED wards respectively. In ICU and MED wards there was a clear and lasting change in the difference between inappropriate to appropriate RBCTs from negative to positive over time with a change-point in the time trend occurring between March 2016 and May 2018 for the ICU ward (the difference between appropriate and inappropriate RBCTs in the ICU ward becomes zero in January 2017), and between May 2017 and July 2018 for the MED ward (the difference between appropriate and inappropriate RBCTs in the MED ward becomes zero in April 2017). In the SURG ward, there is also a sign change in the difference between appropriate and inappropriate RBCTs from negative to positive, that occurs between January 2016 and November 2017 (the difference between appropriate and inappropriate RBCTs in the ICU ward becomes zero in January 2017), however, in this ward the inappropriate RBCTs decrease almost linearly with time from the beginning of the observation period. There was no significant change in the ONC ward over time, but oncology departments are usually quite strict in their transfusion policy and often adhere to a mean hemoglobin level of about 70 g/L in their in-patients. Looking at the average (across medical specialties) difference between appropriate and inappropriate RBCTs in hospitals (Fig. 3), there is a change-point in the time trend of all the hospitals. Particularly, the estimated difference goes from significantly negative to significantly positive in hospitals 1, 3, and 4, while in hospital 2 the difference follows the same trend, but it is not significantly different than zero. In hospital 1, the difference between appropriate and inappropriate RBCTs was significantly negative between July 2014 and November 2016, significantly positive between June 2018 and June 2020, and becomes zero by the end of 2017. In hospital 3, the difference between appropriate and inappropriate RBCTs was significantly negative between March 2014 and July 2016, significantly positive between end of August 2018 and of July 2020, and becomes zero by the end of 2017. In this particular hospital the inappropriate RBCTs decreased linearly with time almost from the beginning of the observation period in 2014. In hospital 4, the difference between appropriate and inappropriate RBCTs was significantly negative between March 2014 and May 2017, significantly positive between July 2018 and April 2021, and becomes zero by the end of 2017.

Because the insurgence of the COVID-19 epidemics impacted majorly on elective and non-urgent hospital activity throughout most of early 2020, we included the time of the first reported cases in our region in the figures to ascertain whether changes in trend were affected by the epidemics. COVID-19 did not significantly affect transfusion practice in a consistent way across specialties or hospital sites.

## Discussion

In our hospital network we observed a significant overall decrease in inappropriate RBCTs in the period October 2017 to March 2021 (35%) compared with the previous period January 2014 to March 2017 (52%) in relation to the publication of new recommendations on transfusion guidelines in 2016. This would appear to be consistent with a positive relationship between guideline publication and change in clinical practice. The specialty with a favorable and persistent trend in the reduction of inappropriate transfusions over time was the internal medicine ward, whereas in the SURG, ONC and ICU wards the change in trend was not as clear-cut or was not observed. Additionally, there were differences in trends within the same medical specialty at different hospital sites, with the MED ward of one of the hospitals driving most of the change, but with a change that commenced well before the introduction of national guidelines. Interestingly, the head of the MED ward at that hospital site was directly involved in drafting the 2016 national guidelines and recommendation on RBCTs^11^, and may have contributed in driving the change in culture, even in the absence of targeted interventions. Because the hospitals are of different size and represent specialties in different areas, this span of data sources enables enhanced generalizability of the findings to a wide class of hospital networks. Since the insurgence of the COVID-19 epidemics impacted majorly on elective and non-urgent hospital activity throughout most of early 2020, which was compounded by issues relating to the supply of blood products during the pandemic, we included the time of the first reported cases in our region in the analysis to ascertain whether changes in trend were affected by the epidemics. COVID-19 did not significantly affect transfusion practice in a consistent way across specialties or hospital sites. The trend to significant change continued after COVID-19, without a tangible inflection on the trend, indicating either a markedly increased or decreased use of inappropriate transfusions.

A strength of the present study is that it uses real-world data that was extracted from a monitoring dashboard and database built without any a priori study plan. Our research project brings about the innovative concept of systematic evaluation and monitoring, using a dashboard, of the RBCTs prescription trends according to the appropriateness of the intervention (threshold of 70 g/L in patients without CVC, and of 80 g/L in patients with CVC). To our knowledge, this approach has never been addressed by other studies in the past^27^, and the threshold of 80 g/L is still largely used for benchmarking purposes even if there is significant scientific evidence supporting a more conservative transfusion strategy^13,28–30^. Another strength of this study is the new way to analyze transfusion data over time as non-linear time series. Indeed, when a high number of covariates may be involved, including observable (like specialties and hospitals) and unobservable (like occurrence of emergency conditions) factors, and whenever no prior information ensures known functional trend in data, standard statistical models may completely fail in capturing the real temporal trend and in identifying an eventual change-point. The introduction of the Generalized Additive Models allows for considering with flexibility and consistency many complex aspects of these data, including autoregressive structure and possible overdispersion.

In 2013 a group of clinicians form our organization championed the Choosing Wisely campaign within our health network and in 2018 the organization committed to join the “Smarter Medicine Choosing Wisely Switzerland” (www.smartermedicine.ch). The group focused on 2 of the 5 key interventions initially promoted by Smarter Medicine, namely the inappropriate use of benzodiazepines and the overprescription of unnecessary blood tests. To tackle those areas the group promoted a series of educational events targeting doctors in order to raise the awareness of the risks in the overprescription of benzodiazepines and in requesting unnecessary blood test. However, the group did not actively promote a campaign to raise the awareness on safe PBM practice across all specialties. Since mentoring is indeed very important in disseminating knowledge and assisting in implementing new guidelines at institutional level^31^, the variable time-points in compliance with the transfusion guidelines suggest that the changes over time in RBCTs usage observed in our health network are due, primarily, if at all, to passive uptake of knowledge from guidelines. Thus, we can infer that if practice guidelines are not accompanied by a targeted education and awareness campaign, their uptake is slow and/or limited. Commencing in January 2021, a specific PBM program that established clear RBCT thresholds and preoperative hemoglobin optimization to reduce both the transfusion rate and index to avoid inappropriate RBCTs was implemented in our hospital network. In the period April 2021 to March 2023 the rate of inappropriate transfusions dropped to 19%, compared to 35% in the period October 2017 to March 2021 (data not shown). The time lag between publication of the transfusion guidelines and the EOC PBM program as a hospital practice policy is due to several factors, primarily, the recognition that the uptake of the guidelines had been slow and variable and that a decision making tool could improve practice change. Guidelines are intended to be more flexible and while they should be followed in most cases, they allow for deviation based on the recognition that, depending on the patient, setting, and other factors, they can be tailored to fit individual needs. On the other hand, policies or standards are more difficult to design because they attempt to make decisions for a collection of patients. They are intended to be applied rigidly and must be followed in virtually all cases. Exceptions will be rare and difficult to justify and therefore many clinical practice guidelines are often not turned into policies. It’s worth noting that our PBM program provides a clinical pathway with a decision making tool rather than a formalized requirement, an approach that has proven to be successful in other areas^32^.

The current data show that a change in practice patterns with a time lag of approximately 2 years following the publication of the Swiss guidelines is consistent with the theory first developed by E. M. Rogers of diffusion of innovation^33^. According to Rogers, innovation diffusion is a five-step process of knowledge, persuasion, decision, implementation, and confirmation^33^. Knowledge from guidelines is only one step towards introducing change^34^. Interestingly, the change over time in the hospital medical ward that drove most of the change in the specialty was linear, starting from 2014, 2 years prior to publication of the Swiss guidelines^11^, but 2 years after the publications of the AABB and ASH guidelines^12,15^. In the US a trend to continued declines in demand for blood products commencing in 2011 was equally observed prior to the introduction of transfusion guidelines^35^. Other studies further demonstrated a continuous decline in the total RBCTs, and transfusions of platelet and plasma units within the last decade^36–39^, as well as a concomitant easing of the economic burden on the healthcare system^10,36^. This raises the question whether guidelines change practice or whether trends in practice change based on evidence are formalized in guidelines. Guidelines translate best evidence into best practice with the aim of promoting quality by reducing healthcare variations, improving diagnostic accuracy, promoting effective therapy, and discouraging ineffective or potentially harmful interventions. Development of guidelines is an in-depth process that requires inputs from many healthcare professionals and evidence should be the keystone in proper guideline development^40^. Our study raises some questions about the general concept of guidelines in clinical practice. Many clinical decisions at the bedside, many medical interventions and rules of operation at hospital are increasingly influenced by guidelines^41^. Over the past decade, clinical guidelines have gradually become a part of clinical practice, although many often use non-systematic methods and are rarely updated in an efficient manner that respect the rapid evolution of biomedical technologies and knowledge^42^. Some of the questions are: is rigid application of guidelines really necessary and helping clinical decision making? What guides guidelines? Are these really determining or following clinical practice?

Another question that arises is whether publishing regional guidelines^11^ several years after the publications of reputable international guidelines^12,15^ is a value-added endeavor. One argument could be that it may reach a broader audience because it overcomes language barriers, where English in not the country’s main language. However, most doctors in non-English speaking countries have some basic literacy in the language and the current ready access to digital information allows for real-time access to the latest information, including the most up-to-date guidelines.

From our analysis we can assume that guidelines are difficult to read and assimilate and their impact is difficult to measure with real world data^43^. They are a consequence, rather than a cause of changes in clinical practice. Indeed, one big problem in producing guidelines is that they are typically realized as a result of randomized trials, which usually study homogeneous populations, answer to very specific questions referred to a predefined cohort of patients, whereas in daily clinical practice the patients present a high degree of heterogeneity which is not included in guidelines. Daily procedures produce guidelines that tend to be published years after, when procedures might already be changed. This study shows, within a completely data-driven approach, that change-point in clinical practice could be considered as predictive of change in guidelines. The case study of RBCTs is likely to be a reflection of the challenges in implementing new clinical guidelines. For instance, the UK National Institute for health and Care Excellence (NICE), which started in 1999, publishes over 20 new clinical practice guidelines every year. In addition to structured educational programs and mentoring, health care organizations need to prioritize the guidelines that are likely to have the largest impact for patients, taking into consideration the sustainability of proposed changes^44^.

We acknowledge some limitations to our study. First, the classification of RBCTs being inappropriately prescribed is based only on the last Hb level recorded pre-transfusion. Therefore it doesn’t take into consideration the dynamic change in Hb prior to RBCT, which in case of severe active bleeding it may classify RBCTs given at Hb levels higher than threshold as inappropriate, while they would in fact be clinically indicated. While this is obviously a shortfall in our analysis, it is unlikely that there was a significant change over time in the proportion of patients with active bleeding that would explain the decline of RBCTs labelled as inappropriate. Second, the use of operational electronic health record data in comparative effectiveness research may be inaccurate, incomplete, transformed in ways that undermine their meaning, of insufficient granularity, and incompatible with research protocols. In our dataset the amount of clinical data used was minimal, and most of the operational data relied on well-defined and traceable parameters, such as Hb level extracted from the laboratory database or transfused blood units extracted from the health record. Data quality and accuracy was previously assessed and validated by two authors (AS and PF) at the time when the reporting platform was created.

In conclusion, our study shows a clear and consistent decrease in inappropriate RBCTs over time between 2014 and 2020 within the entire hospital network, with a decrease in inappropriate RBCTs in the period October 2017 to March 2021 (35%) compared with the previous period January 2014 to March 2017 (52%). The change**-**point in the time trend from more inappropriate to more appropriate RBCTs is observed about 7 years after the publication of the transfusion guidelines by the AABB and almost 2 years following the publication of the Swiss guidelines. The temporal pattern of change in RBCTs practices among hospital sites and specialties was unpredictable. In the absence of a targeted interventions, guidelines alone may not contribute efficiently to rapidly influence medical practice. More structured intervention that include dissemination, education and training, social interaction, decision support systems and standing orders may be necessary to influence clinical practice in accordance to the introduction of new guidelines.

## Supplementary Information

Supplementary Information.

## Data Availability

The dataset used for analysis is available from the corresponding author upon reasonable request.
