# Supplementary material for: Differential impact of transfusion guidelines on blood transfusion practices within a health network
Source: Sci Rep. 2023 Apr 17;13:6264. doi: 10.1038/s41598-023-33549-6 (PMC10109235; doi:10.1038/s41598-023-33549-6)
Supplement: Supplementary file 1 — Supplementary Information. [file 41598_2023_33549_MOESM1_ESM.docx]

# **Statistical Analysis**

In this study, we model time series data collected on the daily counts of RBCTs from several hospitals and different hospital departments. These data are complex and heterogeneous, with inherent characteristics that can distort inferences. For example, it is known that observations in time series data are autocorrelated. Autocorrelation is the [correlation](https://en.wikipedia.org/wiki/Correlation) of a [signal](https://en.wikipedia.org/wiki/Signal_(information_theory)) with a delayed copy of itself as a function of the delay. When modelling time series, failing to account for the serial correlation between successive observations will lead to dependent errors that violates theresidual assumptions. Another important feature that needs to be considered in time series modelling is the time-trend component, which is usually assumed to be linear. While linear trends are sufficient to capture the time variation in some dynamic phenomena, they fail to explain the time-variation in more complex dynamical systems. Last, the outcome that we focus on this study are counts of RBCTs, and the most common statistical model for count data is the Poisson model. The latter, assumes that the mean and the variance of the counts are equal, this assumption however rarely holds with real-life data. Count data in which the variance is greater than the mean, are called “overdispersed”. Overdispersion can be due to unobserved heterogeneity, the presence of outliers, or the excess of zeros in the response variable. When count data are overdispersed, the Poisson distribution is no longer adequate to model the data. Thus, statistical modelling of the daily counts of appropriate and inappropriate RBCTs requires a flexible framework that controls for the abovementioned data characteristics simultaneously. For appropriately modelling the RBCTs time series data, we consider the Generalized Additive Model (GAM) framework.

The GAM approach is a semiparametric modelling technique that allows the outcome to be expressed as a sum of arbitrary functions of the covariates. These functions are called splines and they are constructed as a linear combination of known basis functions. Splines are flexible in the sense that they do not follow any fixed parametric form, but their functional form is estimated from the data. In this work, this is an important feature since we can model (potentially) nonlinear time effects without imposing polynomial forms when modelling the time trends. Moreover, splines can vary across hospitals and hospital departments, which can capture the heterogeneity by fitting different time trends in each RBCT type, hospital, and hospital department. Within a GAM framework, the temporal correlation can be modelled in various ways. Computationally, the most efficient way is to incorporate the autocorrelation directly as a fixed autoregressive effect by including different lag parameters representing the “historical learning”. Alternatively, one can model the autocorrelation by adding a random component that models the autocorrelation though the covariance structure of a Generalized Additive Mixed Model (GAMM). Here, we choose to model the temporal autocorrelation as fixed autoregressive effects in order to reduce the computational complexity. For modelling the overdispersion, count distributions that parametrize the variance separately from the mean such as the negative Binomial or the zero-inflated Poisson are needed.

In this work, we considered different models for count data. Particularly, we modelled the daily RBCT counts using regular Poisson, zero-inflated Poisson, and negative binomial GAMs. Tests for overdispersion and zero inflation were performed on these candidate models in order to determine the distribution that best describes the variability in the data. According to the test results, the negative binomial GAM (nbGAM) was selected as the best fitting to model the RBCT counts. Each observation in the data was then modelled as a function of seven autoregressive effects that correspond to the lagged effects from the previous seven days, a fixed effect for each type of transfusion (appropriate-inappropriate), hospital, and hospital department, and a spline of time for each level of the interaction between type of transfusion, hospital, and hospital department.

## **Statistical model**

Let the response variable $y_{ijkt}$ denote the observed counts of transfusion type $i, i\in\{appropriate, inappropriate\}$, from hospital $j, j\in\{1,..,4\}$, hospital department $k, k\in\{Surgery, ICU, Oncology, Medicine\}$, and day $t, t\in\{01/ 01 / 2014 , \ldots, 23/ 04 / 2021\}$. Moreover, let the transfusion counts from $d$ days before day $t$ to be denoted by $y_{ijkt-d}$. Since the response is a count variable and preliminary exploratory data analyses showed evidence of over-dispersion, we assumed that the response follows a negative binomial distribution. Particularly, we assume that

$y_{ijkt} \sim NB(r_{ijkt}, \pi)$

with mean $E\left( y_{ijkt} \right)=\mu_{ijkt}=\frac{r_{ijkt}}{\pi}$, and variance $V\left( y_{ijkt} \right)= \mu_{ijkt} +\frac{\mu_{ijkt}}{\pi}$ .

In a GAM, the logarithm of the expected value $\mu_{ijkt}$ of the daily transfusion counts is assumed to linearly depend on the transfusion counts up to one week before day $t$, that is $y_{ijkt-1},\ldots, y_{ijkt-7}$, and also to an arbitrary function of time (time trend) with unknown functional form denoted by $f\left( {time}_{ijkt} \right)$. Thus, the model that we consider is the following,

$$\log\left( \mu_{ijkt} \right)=\beta_{0ijk}+ \beta_{1}y_{ijkt-1} +\ldots+ \beta_{7} y_{ijkt-7} +f\left( {time}_{ijkt} \right),$$

Where $\beta_{0ijk}$ corresponds to the intercept of transfusion type , hospital $j$, and department $k$, $\beta_{1}\ldots\beta_{7}$ correspond to the linear autoregressive effects of one, two, …, and seven days ago, and the function of time $f\left( {time}_{ijkt} \right)$is a centred twice-differentiable smooth function.
